# Integrative Transcriptomic and Metabolomic Analysis Reveals the Molecular Mechanism of Red Maple (*Acer rubrum* L.) Leaf Coloring

**DOI:** 10.3390/metabo13040464

**Published:** 2023-03-23

**Authors:** Yuanyuan Luo, Min Deng, Xia Zhang, Damao Zhang, Wenqi Cai, Yuelin Long, Xingyao Xiong, Yanlin Li

**Affiliations:** 1 College of Horticulture, Hunan Agricultural University, Changsha 410128, China; 2 College of Oriental Science & Technology, Hunan Agricultural University, Changsha 410128, China; 3 College of Agronomy, Hunan Agricultural University, Changsha 410128, China; 4 Engineering Research Center for Horticultural Crop Germplasm Creation and New Variety Breeding, Ministry of Education, Changsha 410128, China; 5 Hunan Mid-Subtropical Quality Plant Breeding and Utilization Engineering Technology Research Center, Changsha 410128, China; 6 College of Landscape Architecture and Art Design, Hunan Agricultural University, Changsha 410128, China; 7 Agricultural Genomics Institute at Shenzhen, Chinese Academy of Agricultural Sciences, Shenzhen 518120, China; 8 Institute of Vegetables and Flowers, Chinese Academy of Agricultural Sciences, Beijing 100081, China; 9 Kunpeng Institute of Modern Agriculture, Foshan 528200, China; 10 School of Biological Sciences, Nanyang Technological University, 60 Nanyang Drive, Singapore 637551, Singapore

**Keywords:** *Acer rubrum* L., transcriptomics, metabolomics, flavonoid, coloring

## Abstract

This study employed a combination of ultraviolet spectrophotometry, LC-ESI-MS/MS system, and RNA-sequencing technology; the extracts and isolation of total RNA from the red and yellow leaf strains of red maple (*Acer rubrum* L.) at different developmental stages were subjected to an intercomparison of the dynamic content of chlorophyll and total anthocyanin, flavonoid metabolite fingerprinting, and gene expression. The metabonomic results indicated that one hundred and ninety-two flavonoids were identified, which could be classified into eight categories in the red maple leaves. Among them, 39% and 19% were flavones and flavonols, respectively. The metabolomic analysis identified 23, 32, 24, 24, 38, and 41 DAMs in the AR1018r vs. AR1031r comparison, the AR1018r vs. AR1119r comparison, the AR1031r vs. AR1119r comparison, the AR1018y vs. AR1031y comparison, the AR1018y vs. AR1119y comparison, and the AR1031y vs. AR1119y comparison, respectively. In total, 6003 and 8888 DEGs were identified in AR1018r vs. AR1031r comparison and in the AR1018y vs. AR1031y comparison, respectively. The GO and KEGG analyses showed that the DEGs were mainly involved in plant hormone signal transduction, flavonoid biosynthesis, and other metabolite metabolic processes. The comprehensive analysis revealed that caffeoyl-CoA 3-*O*-methyltransferase (Cluster-28704.45358 and Cluster-28704.50421) was up-regulated in the red strain but down-regulated in the yellow strain, while Peonidin 3-*O*-glucoside chloride and Pelargonidin 3-*O*-beta-*D*-glucoside were up-regulated in both the red and yellow strains. By successfully integrating the analyses on the behavior of pigment accumulation, dynamics of flavonoids, and differentially expressed genes with omics tools, the regulation mechanisms underlying leaf coloring in red maple at the transcriptomic and metabolomic levels were demonstrated, and the results provide valuable information for further research on gene function in red maple.

## 1. Introduction

Plants are essential to urban landscapes and can improve living environmental decoration [1]. Urban trees substantially contribute to the quality of urban living [2]. The genus *Acer* (Aceraceae) is an essential ornamental plant for landscapes and includes approximately 129 species that primarily grow in the northern hemisphere [3]. Red maples (*Acer rubrum* L.), a critical member of the genus Acer, are widely grown in parks, urban spaces, and gardens in East Asia and North America due to their high ornamental value [3,4]. October Glory (*Acer rubrum* L., red maple) from the northeastern United States was introduced to China in 2011 [5]. Today, red maple is one of the most popular landscape trees planted in China because it features ideal colorful leaves.

People like plants with colored leaves because such plants show different colors in different seasons. Why do plant leaves have different colors? The presentation of changes in colors in plants is very complex and is determined by their metabolic composition [4]. Betalains, carotenoids, and anthocyanins, which are the primary groups of pigments, create an attractive natural display of flower colors. Anthocyanins are a group of water-soluble pigments in plants, and their biosynthesis and regulation are well understood due to their broad distribution [6]. Anthocyanins are compound flavonoids that create multiple tissues of different colors in flowers, leaves, vegetables, fruits, seeds, etc. [7].

Anthocyanidins are plant pigments that form and accumulate anthocyanins in vacuoles through conjugation with sugar molecules and can be classified mainly into flavonoids and phenolics. Anthocyanins can also be divided into anthocyanidin derivatives, nonacylated anthocyanidin glucoside, and acylated anthocyanidin glucoside [8]. More than 635 anthocyanins have been identified in various flowers, vegetables, leaves, and fruits [9]. To date, only eight metabolites associated with the synthesis and accumulation of anthocyanins have been identified in *Acer* plants, and related substances have not been identified in *A. rubrum* [4]. The core compounds of anthocyanidin derivatives in plants include cyanidin (Cy), delphinidin (Del), pelargonidin (Pel), peonidin (Peo), petunidin (Pet), and malvidin (Mal) [10].

Anthocyanins are synthesized via the phenylpropanoid and flavonoid biosynthetic pathways [11]. Many constructive genes are involved in anthocyanin biosynthesis. Some of these genes have been cloned in different plants, including chalcone synthase (*CHS*), dihydroflavonol 4-reductase (*DFR*), phenylalanine ammonia-lyase (*PAL*), chalcone isomerase (CHI), 4-coumarate–coenzyme a ligase (*4CL*), flavanone 3-hydroxylase (*F3H*), and anthocyanidin synthase (*ANS*) [12,13,14,15,16,17,18,19,20,21,22]. However, limited research has sought to elucidate the molecular mechanisms of pigment synthesis in red maple [4,23]. With the development of omics technologies, increasingly more research is being performed using such pigments, especially in complex genomes and perennials. Transcriptomic and metabolomic technologies are the most commonly used methods to determine the regulation mechanisms of stress [24,25,26,27,28], development [29,30,31,32], complex traits [33,34,35], metabolite biosynthesis [34,36,37] and coloring [7,8,9,11,38]. Using transcriptomic and metabolomic technologies, non-structural carbohydrates [39], photosynthetic pigments [23], hormone pathways [40], and pigmentation [4] were investigated in *Acer rubrum* L. during developmental leaf senescence. The analysis partly explained the mechanism of red maple leaf color formation. However, this mechanism must still be clarified for red maple.

To investigate the coloring mechanism of red maple, we performed metabolomic and transcriptomic analysis of red maple leaves obtained during different developmental stages. In addition, we investigated the transcript profiles and metabolite levels of flavonoids and identified differentially expressed genes (DEGs) and differentially accumulated metabolites (DAMs) involved in flavonoid biosynthesis. The multi-omics of combining pigment phenotypic analysis and flavonoid metabolomic and transcriptomic analyses could better discover the mechanism of leaf color changes in red maple during different development stages. Additionally, the results of this comprehensive research could help increase our understanding of the gene regulation and metabolic mechanisms in red maple. Moreover, these results could provide the basis for color improvement using advanced breeding technology in red maple and other colored foliage plants.

## 2. Materials and Methods

### 2.1. Plant Materials and Culture Conditions

*Acer rubrum* strains (‘October Glory-1’ and ‘October Glory-2’) were obtained from the Hunan Agricultural University, Changsha, Hunan province, People’s Republic of China (E 113.08, N 28.18). We selected 12-year-old *A. rubrum* plants as the test objects. The plant materials were planted on both roadsides in a natural environment. The leaf color of ‘October Glory-1’ is red in late autumn (ARr), and that of ‘October Glory-2’ is yellow in late autumn (ARy). The leaves were collected from the red and yellow strains at three different times, including on 18 October 2018 (AR1018r or AR1018y); 31 October 2018 (AR1031r or AR1031y); and 19 November 2018 (AR1119r or AR1119y). In this study, 30 leaves were collected for each biological sample, and each leaf color collected was repeated in three biological replicates. All the samples were stored in a cryogenic freezer at −80 °C.

### 2.2. Measurement of Chlorophyll Content

Chlorophyll extraction and detection were modified according to the method described by Arnon [41]. The red maple leaves (0.2 g) were washed with distilled water and dried with clean absorbent paper. Each sample was clipped into strips (about 0.2 cm in width) with scissors. Then, the strips were transferred into a new clean 15 mL tube filled with 10 mL of alcohol at a concentration of 95% at 4 °C and left in the dark for at least 24 h until the strips were immersed and bleached. Each treatment used three independent biological replicates. The absorbance value of each supernatant from the treatment was detected using an ultraviolet spectrophotometer. The extracted supernatant was analyzed immediately. The absorbance values at different wavelength of 470, 649, and 665 nm were measured with an ultraviolet spectrophotometer (AOE TSD-599, Shanghai, China), and each colored-leaf variety had six replicates.

### 2.3. Measurement of Anthocyanin Content

Anthocyanin content was detected based on the pH differential method for cyanidin-3-glucoside content in leaves. The minor adjustments made to the test method by Zhang et al. was adopted [42] with the following calculation formula:
Ca = 13.95A665 − 6.88649;Cb = 24.96A649 − 7.32A665;Cc = (1000A470 − 2.05Ca − 114.8Cb)/248;TA = A × MW × 5 × 100 × V/e,
where TA stands for total anthocyanin content (mg 100 g^−1^, as cyanidin-3-O-glucose equivalent), V stands for final volume (mL), and A = [A510 (pH 1.0) − A700 (pH 1.0)] − [A510 (pH 4.5) − A700 (pH 4.5)]. A molar absorptivity (e) of 26,900 m^2^.mol^−1^ and a molecular weight (MW) of 449.2 Da were used according to Wrolstad et al. (1982). Three measurements were taken for every six biological replicates [43].

### 2.4. Sampling Preparation and Metabolite Extraction

The leaves were collected from the red and yellow strains at three different times, including on 18 October 2018 (AR1018r or AR1018y); 31 October 2018 (AR1031r or AR1031y); and 19 November 2018 (AR1119r or AR1119y). The flavonoid extraction method was performed following the protocol of previously published documents [44,45,46]. The leaves were frozen in liquid nitrogen immediately, transferred to the refrigerator, and stored at −80 °C until further analysis. The freeze-dried leaves were crushed using a mixer mill (MM 400, Retsch) with zirconia beads for 1.5 min at 30 Hz. Next, 100 mg of the powder was weighed and extracted overnight at 4 °C with 1.0 mL of 70% aqueous methanol. Following centrifugation at 10,000× *g* for 10 min, the extracts were absorbed by CNWBOND Carbon-GCB SPE Cartridge (ANPEL, Shanghai, China), with the size of 250 mg and 3 mL, and then filtrated by SCAA-104 (ANPEL, Shanghai, China) with the size of 0.22 μm pore before the LC/MS analysis. 

### 2.5. Metabolite Profiling

The sample extracts were analyzed using an LC-ESI-MS/MS system of HPLC with Shim-pack UFLC SHIMADZU CBM30A system (Shimadzu Corporation, Kyoto, Japan) and MS with Applied Biosystems 6500 Q TRAP (Shimadzu Corporation, Kyoto, Japan). The analytical conditions were as follows: HPLC column with waters ACQUITY UPLC HSS T3 C18 (1.8 µm, 2.1 × 100 mm); solvent system with water (0.04% acetic acid)/acetonitrile (0.04% acetic acid); gradient program was 100:0 *v*/*v* for 0 min, 5:95 *v*/*v* for 11.0 min, 5:95 *v*/*v* for 12.0 min, 95:5 *v*/*v* for 12.1 min, and 95:5 *v*/*v* at 15.0 min; flow rate was 0.40 mL min^−1^; temperature was 40 °C; and injection volume was 2 μL. The effluent was alternatively connected to an ESI-triple quadrupole–linear ion trap (Q TRAP)-MS.

The linear ion trap (LIT) (Shimadzu Corporation, Kyoto, Japan) and triple quadrupole (QQQ) (Shimadzu Corporation, Kyoto, Japan) scans were acquired on a triple quadrupole–linear ion trap mass spectrometer (Q TRAP) (Shimadzu Corporation, Kyoto, Japan), API 6500 Q TRAP LC/MS/MS System, equipped with an ESI Turbo Ion–Spray interface, operating in a positive ion mode and controlled using the Analyst 1.6.3 software (AB Sciex). The ESI source operation parameters were as follows: ion source was turbo spray; source temperature was 500 °C; and ion spray voltage (IS) was 5500 V. The ion source gas I (GSI), gas II (GSII), and curtain gas (CUR) were set at 55, 60, 21, and 25.0 psi, respectively. The collision gas (CAD) was set as high. Instrument tuning and mass calibration were performed with 10 and 100 μmol L^−1^ polypropylene glycol solutions in the QQQ and LIT modes, respectively. The QQQ scans were acquired as MRM experiments with collision gas (nitrogen) set to 5 psi. The DP and CE for individual MRM transitions were performed with further DP and CE optimization. A specific set of MRM transitions was monitored for each period according to the metabolites eluted within the period. Further mass spectrometric methods were performed following the published protocols of Chen et al. (2013) [47] and Zhu et al. (2018) [48].

### 2.6. Identification of Metabolites 

The identified metabolites were annotated using the KEGG compound database [49]. Differentially accumulated metabolites (DAMs) were defined as those that exhibited a fold change ≥ 2 or a fold change ≤ 0.5 and a variable importance in project (VIP) value ≥ 1 between pairwise comparisons. Principal component analysis (PCA) and partial least squares discriminant analysis (PLS-DA) were performed using SIMCA 14.1.

### 2.7. RNA-Seq and Functional Annotation

The leaves were collected from the red and yellow strains at two different times: 18 October 2018 (AR1018r or AR1018y) and 31 October 2018 (AR1031r or AR1031y). RNA purity was assessed using a NanoPhotometer^®^ spectrophotometer (Thermo Fisher, Waltham, MA, USA). The RNA concentration was measured using a Qubit^®^ RNA Assay Kit in a Qubit^®^ 2.0 Fluorometer (Life Technologies, Carlsbad, CA, USA). RNA integrity was assessed using an RNA Nano 6000 Assay Kit in an Agilent Bioanalyzer 2100 system (Agilent Technologies, Santa Clara, CA, USA). A total amount of 1.5 µg RNA per sample was used as the input material for the RNA sample preparations. Sequencing libraries were generated using the NEBNext^®^ Ultra™ RNA Library Prep Kit for Illumina^®^ (NEB, Ipswich, CA, USA) following the manufacturer’s recommendations, and index codes were added to attribute the sequences to each sample. Briefly, mRNA was purified from total RNA using poly-T oligo-attached magnetic beads. Fragmentation was carried out using divalent cations under an elevated temperature in a NEBNext First Strand Synthesis Reaction Buffer (5×). First-strand cDNA was synthesized using a random hexamer primer and M-MuLV Reverse Transcriptase (RNase H-). Second-strand cDNA synthesis was subsequently performed using DNA Polymerase I and RNase H. The remaining overhangs were converted into blunt ends via exonuclease/polymerase activities. After adenylation of the 3′ ends of DNA fragments, the NEBNext Adaptor with a hairpin loop structure was ligated to prepare for hybridization. 

To select preferential cDNA fragments with 250~300 bp in length, the library fragments were purified with an AMPure XP system (Beckman Coulter, Beverly, USA). Then, 3 µL of USER Enzyme (NEB, USA) was used with size-selected, adaptor-ligated cDNA at 37 °C for 15 min, followed by 5 min at 95 °C, before conducting PCR. Then, PCR was performed with Phusion High-Fidelity DNA polymerase, Universal PCR primers, and an Index (X) primer. Lastly, the PCR products were purified (AMPure XP system), and the library quality was assessed on an Agilent Bioanalyzer 2100 system. The raw sequence data reported in this paper were deposited in the Genome Sequence Archive (Genomics, Proteomics & Bioinformatics 2021) of the National Genomics Data Center (Nucleic Acids Res 2022), China National Center for Bioinformation/Beijing Institute of Genomics, Chinese Academy of Sciences (GSA: CRA009292); the data are publicly accessible at https://ngdc.cncb.ac.cn/gsa (accessed on 14 February 2023).

The raw data (raw reads) in the fastq format were first processed through in-house Perl scripts. In this step, clean data (clean reads) were obtained by removing reads containing an adapter, reads containing ploy-N, and low-quality reads from the raw data. At the same time, the Q20, Q30, and GC content, as well as the sequence duplication levels, of the clean data were calculated. All downstream analyses were based on clean data of high quality.

Gene function was annotated based on Nr (NCBI non-redundant protein sequences), Nt (NCBI non-redundant nucleotide sequences), Pfam (protein family), KOG/COG (clusters of orthologous groups of proteins), Swiss-Prot (a manually annotated and reviewed protein sequence database), KO (KEGG Ortholog database), and GO (Gene Ontology) databases.

### 2.8. Analysis of Differentially Expressed Gene (DEG), GO, and KEGG Enrichment

For the differentially expressed genes (DEGs) of the red maple leaves during different developmental stages, the criterion of *padj* (*p*-value was adjusted) < 0.05 and |log2 (fold change)| > 1 was used. The differential expression analysis of two samples was performed using the DEGseq R package (1.10.1). The resulting *p*-values were adjusted using the approach by Benjamini and Hochberg for controlling the false discovery rate. Genes with an adjusted *p*-value < 0.05 found by DESeq were assigned as differentially expressed.

Gene Ontology (GO) enrichment analysis of the differentially expressed genes (DEGs) was implemented using the GOseq R packages based on Wallenius’s non-central hyper-geometric distribution [50], which can adjust for gene length bias in DEGs. The identified genes were annotated using the KEGG database [51]. We used the KOBAS [52] software to test the statistical enrichment of differentially expressed genes in the KEGG pathways.

### 2.9. Integrative Analysis of Metabolomic and Transcriptomic Data

For the combined analysis of metabolomic and transcriptomic data, we conducted a correlation analysis between the DEGs and DAMs mapped onto the flavonoid component biosynthesis pathways in the red and yellow strains. The Pearson correlation coefficients (PCCs) of the DEGs and DAMs were calculated using the R package. We constructed a gene–metabolite network map based on the Pearson correlation coefficients (with a correlation coefficient >0.9 or <0.9) and *p*-value (with a *p*-value < 0.05) using Cytoscape v3.2.0. 

## 3. Results and Discussion

### 3.1. Anthocyanin and Chlorophyll Content in Red Maple Leaves

**Figure 1:**
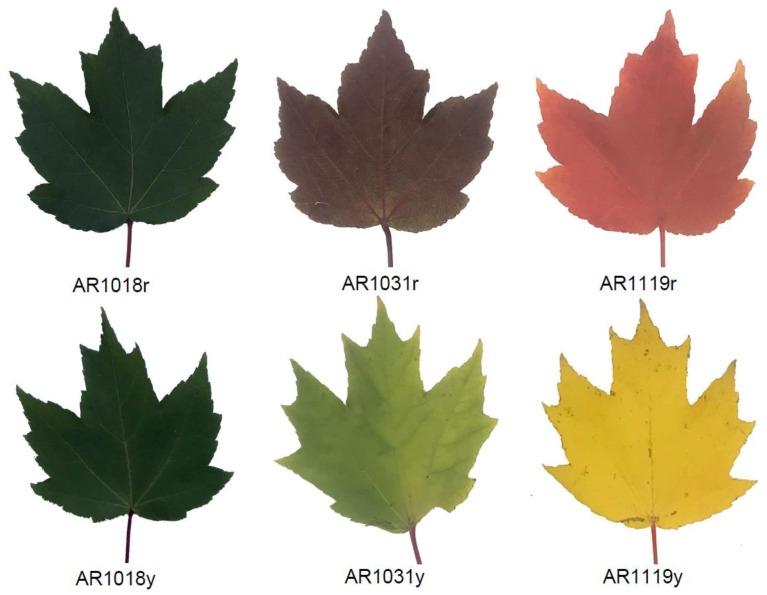
The leaves used in this study were from red and yellow strains. Note: the leaves were collected from red and yellow strains at three different times, including on 18 October 2018 (AR1018r or AR1018y); 31 October 2018 (AR1031r or AR1031y); and 19 November 2018 (AR1119r or AR1119y), respectively.

**Figure 2:**
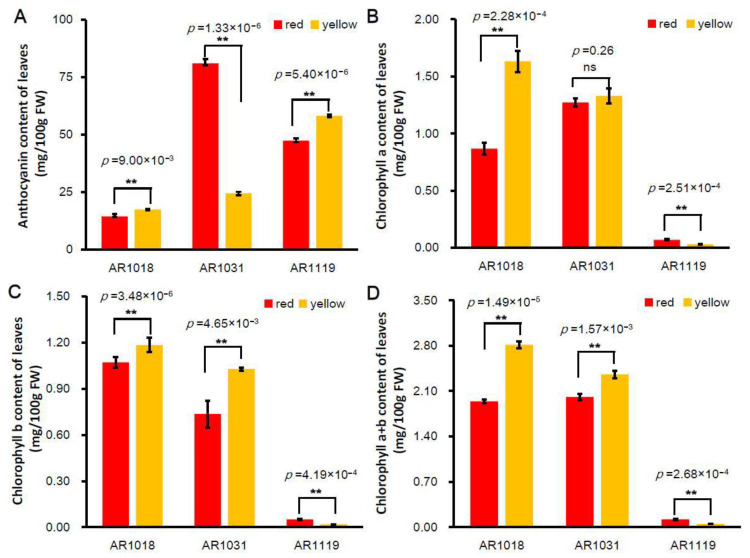
Pigment content of differently colored leaves in red maple. Note: (**A**) anthocyanin content, (**B**) chlorophyll-a content, (**C**) chlorophyll-b content, and (**D**) chlorophyll-a + b content (averages ± standard errors; ** *p* < 0.01; ns: not significant).

Pigment anthocyanidins are widely distributed in flowers, vegetables, leaves, and fruits [8]. Anthocyanin is the vital pigment in yellow and red leaves of red maples [23,53]. Two *Acer rubrum* strains (′October Glory-1′ and ′October Glory-2′, with red and yellow leaves, respectively) were used in this study (Figure 1). To investigate the biosynthesis of pigments in red maple leaves, the alcohol extraction method was used to measure the content of total anthocyanin and chlorophyll in differently colored leaves. The results showed that the content of total anthocyanins in the red strain first increased and then decreased. In contrast, the anthocyanin content presented an increasing tendency in the yellow strain. The content of anthocyanins in the yellow strain was significantly higher than that of the red strains in AR1018 (18 October) and AR1119 (19 November) and significantly lower than that in AR1031 (31 October) (Figure 2A).

Chlorophyll, carotenoids, and flavonoids are known as the major pigments of color in plant tissues [54]. The degree of chlorophyll degradation is strongly correlated with anthocyanins [55]. The chlorophyll-a content showed a similar tendency in the red strain. However, the chlorophyll-a content showed a decreasing tendency in the yellow strain, and the chlorophyll-a content in the red strain was significantly lower than that of the yellow strain in AR1018 but significantly higher than that of the yellow strain in AR1119 (Figure 2B). Subsequently, the chlorophyll-b content presented a decreasing trend in the red and yellow strains. The chlorophyll-b content in the red strain was significantly lower than that in the yellow strain in AR1018 and AR1031 but significantly higher than that in AR1119 (Figure 2C). Finally, the chlorophyll-a + b content displayed a similar tendency to chlorophyll-a (Figure 2D). These results indicate that the contents of total anthocyanin, chlorophyll-a, chlorophyll-b, and chlorophyll-a + b of the red and yellow strains leaves exhibited significant differences during different developmental stages (Figure 1 and Figure 2). Additionally, this was constant during the senescence of leaves in autumn [55].

### 3.2. Identification and Quantification of Flavonoid Components in Red Maple

In order to study the variations in flavonoids in different strains of red maple during different developmental stages of leaf senescence, we performed flavonoid profiling on samples of natural maple that were aged twelve years. Briefly, 192 flavonoids were detected. These flavonoids could be classified into anthocyanins, flavonols, flavanones, flavonoids, flavones, isoflavones, polyphenols, and proanthocyanidins (eight categories) (Supplemental Table S1). Among them, 39% (74) and 19% (36) in red maple leaves were flavones and flavonols, respectively. These results showed that flavones and flavonols were the primary flavonoid components in red maple leaves.

**Figure 3:**
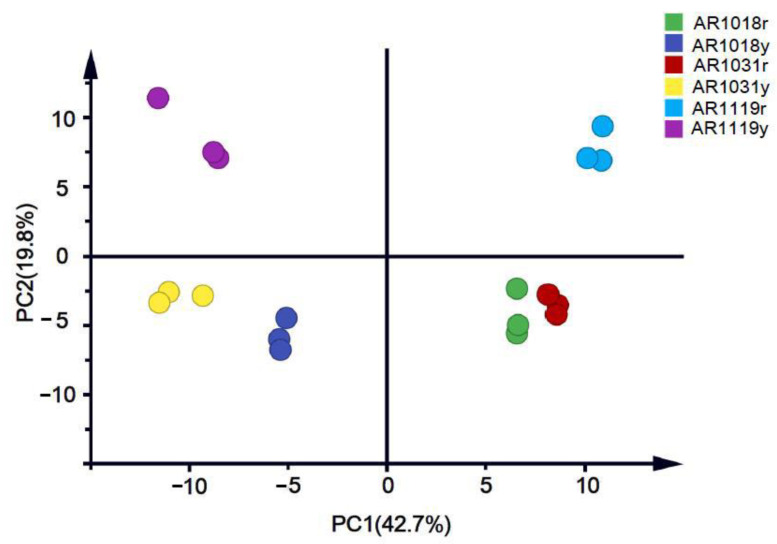
Metabolite principal component analysis of red maple leaves.

**Figure 4:**
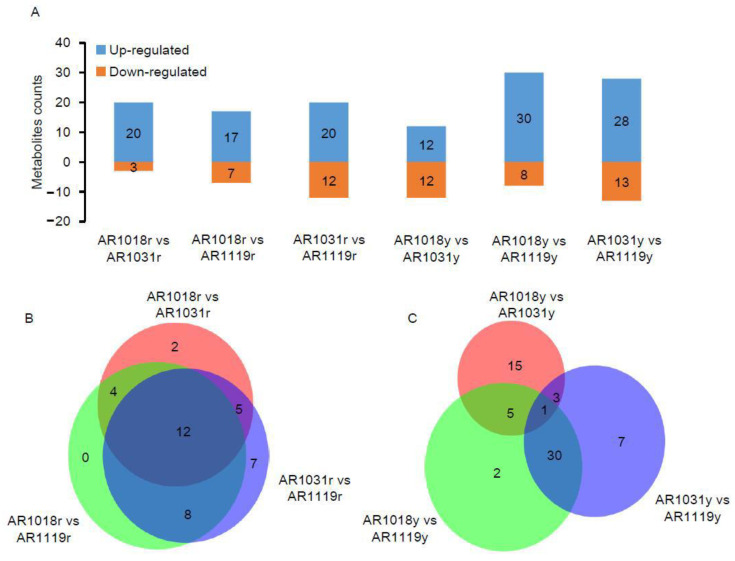
The differentially accumulated metabolite analysis. Note: (**A**) the number of DAMs in three groups. (**B**) Venn diagram of red-strain DAMs in three groups. (**C**) Venn diagram of yellow-strain DAMs in three groups.

Then, we performed a principal component analysis (PCA) on the data to better understand the overall flavonoid metabolome (Figure 3). The PC1 and PC2 explained 42.7% and 19.8% of the metabolite variations among all the samples, respectively. The red and yellow strains could be separated into different groups. Moreover, the samples from different developmental stages were well distinguished (Figure 4).

### 3.3. Identified Differential Accumulation of Metabolites Involved in Flavonoid Biosynthesis

To date, metabolomics has been used to determine the mechanisms underlying color formation in tea [8,36,38], asparagus [7], potato [9], cucumber [11], chokecherry [56], and red maple [23]. Previous studies were performed on non-structural carbohydrates [39], photosynthetic pigments [23], hormone pathways [40], and pigmentation [4] in *Acer rubrum* L. during developmental leaf senescence. In order to determine the variation in metabolite abundance during developmental leaf senescence, differentially accumulated metabolites (DAMs) were defined as those that exhibited a fold change ≥ 2 or a fold change ≤ 0.5 and a variable importance in project (VIP) value ≥ 1 between pairwise comparisons. Using the OPLS-DA model and statistical analysis, the metabolomic analysis identified 23 DAMs, including 20 up- and 3 down-regulated metabolites in AR1018r compared to AR1031r. In the comparison of AR1018r to AR1119r, 32 DAMs, including 20 up- and 12 down-regulated metabolites, were identified. In the AR1031r vs. AR1119r comparison group, 24 DAMs, including 17 up- and 7 down-regulated metabolites, were identified (Figure 4A,B).

Meanwhile, in the AR1018y vs. AR1031y comparison group, 24 DAMs, including 12 up- and 12 down-regulated metabolites, were identified. In the AR1018y vs. AR1119y comparison group, 38 DAMs, including 30 up- and 8 down-regulated metabolites, were identified. In the AR1031y vs. AR1119y comparison group, 41 DAMs, including 28 up- and 13 down-regulated metabolites, were identified (Figure 4A,C). Furthermore, 12 DAMs, 7 anthocyanins, 2 flavones, and 2 flavonols were identified, all in the AR1018r vs. AR1031r and AR1031r vs. AR1119r comparison groups (Figure 4B). Furthermore, only C-pentosyl apigenin O-salicyloyl hexoside was identified in the AR1018y vs. AR1031y and AR1031y vs. AR1119y comparison groups (Figure 4C, Supplemental Table S2). This flavone was massively accumulated in the AR1119yr- and AR1119y-colored leaves of the two strains of red maple. These results indicated that anthocyanin (especially Peonidin 3-*O*-glucoside chloride and Pelargonidin 3-*O*-beta-*D*-glucoside), flavone, and flavonol play essential roles in the red and yellow strains of red maple leaf coloring, respectively.

### 3.4. KEGG Pathway Mapping of DAMs

**Figure 5:**
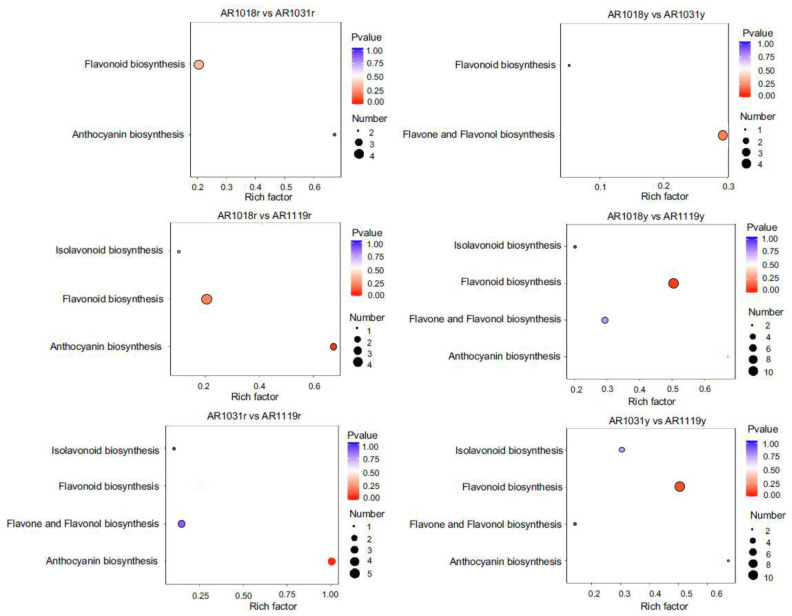
KEGG pathway analysis of DAMs in different groups.

KEGG pathway analysis was performed to determine which cellular pathways might be enriched for the differentially accumulated metabolites in different samples. The metabolite changes were primarily associated with anthocyanin, flavonoid, isoflavonoid, flavone, and flavonol biosynthesis, suggesting that these metabolic pathways play an essential role in the leaf coloring of red maple (Figure 5 and Figure S1). The details for the differential flavonoid component for all comparison groups are shown in Supplemental Table S2. The DAMs between AR1018r and AR1031r, between AR1018r and AR1119r, and between AR1031r and AR1119r of the red strain were all found to be mainly co-enriched in anthocyanin and flavonoid biosynthesis. In contrast, the DAMs between AR1018y and AR1031y, between AR1018y and AR1119y, and between AR1031y and AR1119y of the yellow strain were all found to be mainly co-enriched in flavonoid, flavone, and flavonol biosynthesis (Figure 5 and Figure S1, Supplemental Table S3). These results showed that the metabolic pathways related to anthocyanin and flavonoid changed significantly with leaf development in the red stain, while flavonoid, flavone, and flavonol changed significantly with leaf development in the yellow stain and showed different accumulation patterns.

### 3.5. Sequencing Quality Statistics

**Figure 6:**
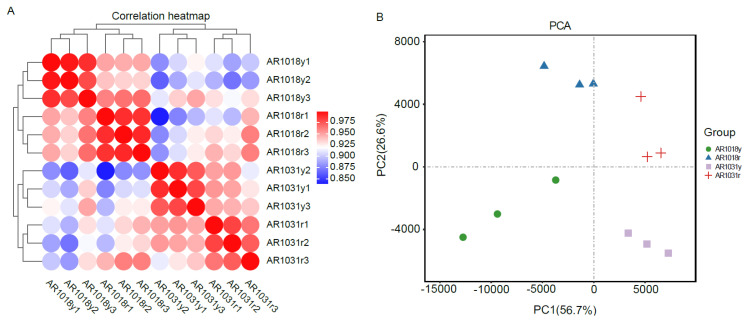
The Pearson correlations between each sample (**A**) and principal component analysis of red maple leaves (**B**).

Twelve libraries of red maple in the two leaf developmental stages were sequenced using the Illumina paired-end sequencing method with three biological replicates per stage, yielding 41 to 62 million high-quality clean reads, with 53 million clean reads per library on average. An overview of the RNA-seq data for the twelve libraries is shown in Table 1. In total, 51,556,563, 56,453,487, 49,761,007, and 55,327,094 clean reads for AR1018y, AR1018r, AR1031y, and AR1031r were produced, respectively, via RNA-seq on the Illumina Hiseq platform, and 639.29 million high-quality clean reads were obtained from the 12 libraries. Each sample′s average GC content was 43.13%. The Q30 ranged from 90.43% to 92.59%, with an average of 91.91%. Ultimately, 103,829 unigenes were assembled from the 12 libraries (Table 1). The Pearson correlations among the AR1018r, AR1018y, AR1031r, and AR1031y replicates ranged from 0.90 to 1 (Figure 6A). The PCA showed that AR1018r, AR1018y, AR1031r, and AR1031y were separately aggregated. The PC1 and PC2 explained 56.7% and 26.6% of the gene expression variations among all the samples, respectively, indicating striking differences in gene expression profiles (Figure 6B).

### 3.6. Differentially Expressed Genes Identified

**Figure 7:**
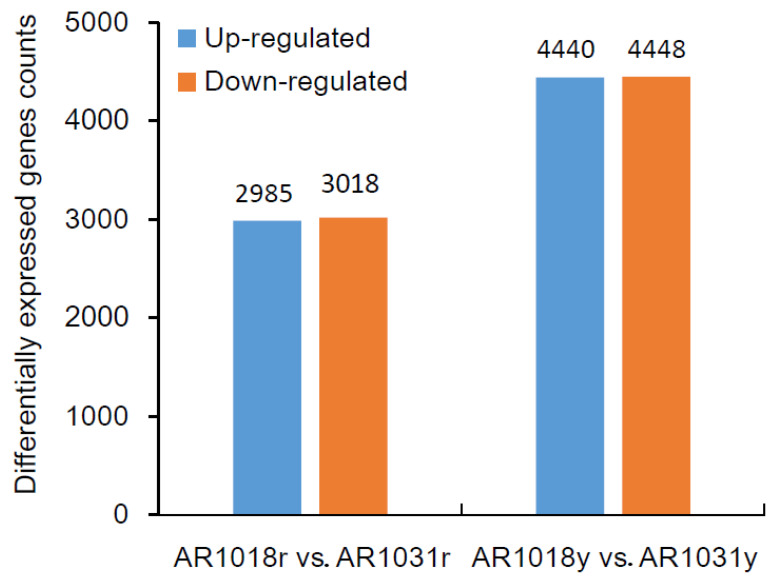
The differentially expressed genes among the groups for the red and yellow strains.

To identify differentially expressed genes (DEGs) of the red maple leaves at different developmental stages, the criteria of padj (adjusted *p*-value) < 0.05 and |log2 (fold change)| > 1 were used. The differential expression analysis of the two samples was performed using the DEGseq R package. A total of 6003 DEGs were identified between AR1018r and AR1031r, with 2985 and 3018 genes being up-regulated and down-regulated in AR1031r, respectively. In total, 8918 DEGs were identified between AR1018y and AR1031y. Additionally, 4440 genes were up-regulated and 4448 genes were down-regulated in AR1031y (Figure 7, Supplemental Table S3).

### 3.7. GO and KEGG Analysis of DEGs

**Figure 8:**
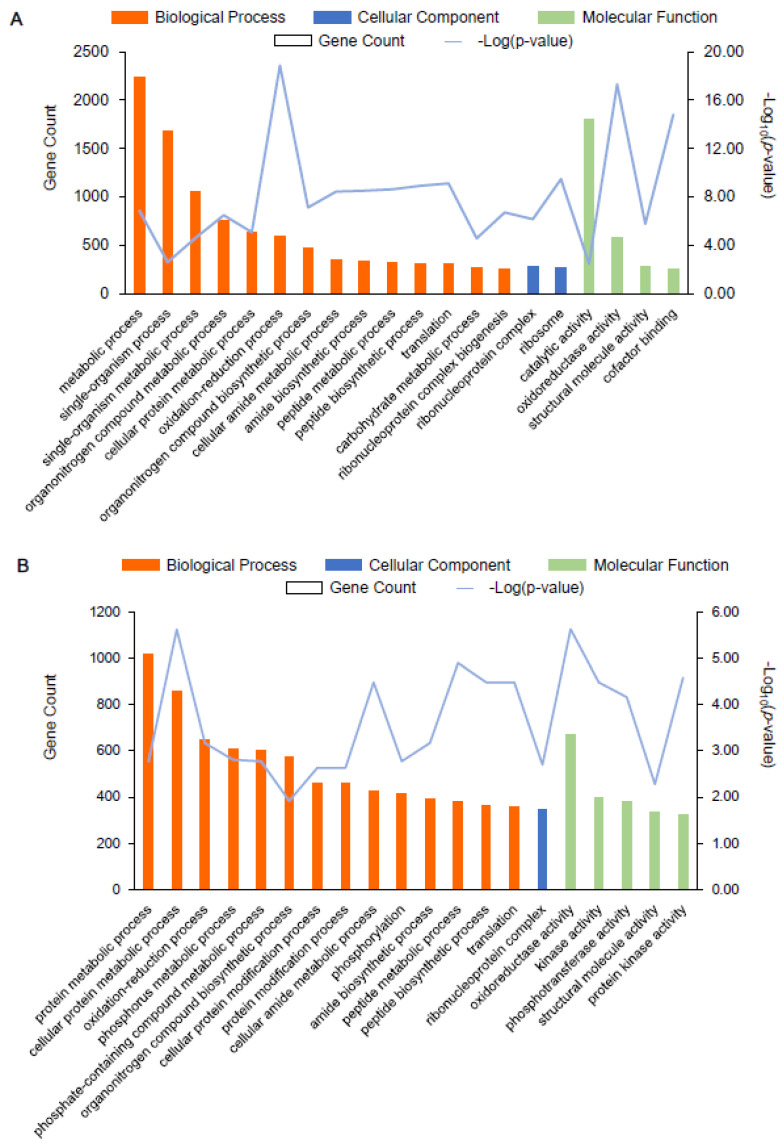
GO enrichment analysis of the top 20 most strongly represented categories. Note: the degree of GO enrichment is represented by the –log10 (*p*-value) (line chart) and the amount of DEG (column chart) enrichment in each category. (**A**) represents AR1018r vs. AR1031r, and (**B**) represents AR1018y vs. AR1031y.

Next, we annotated the GO functional enrichment analysis of the DEGs between AR1018r and AR1031r and between AR1018y, and AR1031y. The DEGs were divided into molecular function, biological process, and cellular component categories. Overall, 2104, 949, and 456 unique GO items were assigned to the biological process, molecular function, and cellular component terms, respectively, in the AR1018r vs. AR1031r comparison. There were 44 GO classification subcategories. The top 20 enrichment classifications are shown in Figure 6A. The genes in the biological process category were primarily matched and classified into metabolic processes, single-organism processes, and single-organism metabolic processes. Most of the unigenes exhibited catalytic activity, oxidoreductase activity, and structural molecular activity in the molecular function term. The most abundant GO terms in the cellular component category included ribonucleoprotein complex and ribosome (Figure 8A, Supplemental Table S4). These biological processes, cellular components, and molecular functions were found to play essential roles in the coloring of red-strain leaves.

In total, 2311, 1048, and 528 unique GO items were assigned to the biological process, molecular function, and cellular component terms in the AR1018y vs. AR1031y comparison, respectively. There were 32 GO classification subcategories, and the top 20 enrichment classifications are shown in Figure 8B. The genes in the biological process category were primarily matched and classified into protein metabolic processes, cellular protein metabolic processes, and oxidation-reduction processes. In the molecular function term, most of the unigenes exhibited oxidoreductase activity, kinase activity, and phosphotransferase activity. The most abundant GO terms in the cellular component category included ribonucleoprotein complex (Figure 8B, Supplemental Table S4). These biological processes, cellular components, and molecular functions played essential roles in the coloring of yellow-strain leaves.

**Figure 9:**
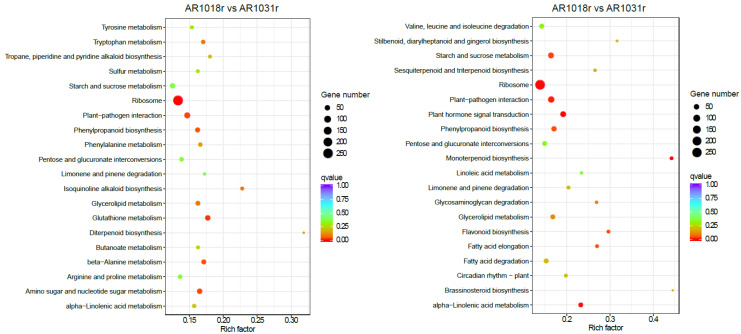
Kyoto Encyclopedia of Genes and Genomes (KEGG) pathway analysis of the DEGs.

In the KEGG pathway-enrichment analysis, matches were found for 2119 and 2287 unigenes, which mapped onto 113 and 117 KEGG pathways in the AR1018r vs. AR1031r comparison and the AR1018y vs. AR1031y comparison, respectively. According to the KEGG pathway database, the main enriched metabolic processes were ribosome, glutathione metabolism, amino sugar and nucleotide sugar metabolism, beta-alanine metabolism, and phenylpropanoid biosynthesis in the AR1018r vs. AR1031r comparison; the main enriched metabolic processes were monoterpenoid biosynthesis, plant hormone signal transduction, ribosome, plant–pathogen interaction, phenylpropanoid biosynthesis, alpha-linolenic acid metabolism, and flavonoid biosynthesis in the AR1018y vs. AR1031y comparison (Figure 9).

### 3.8. Integrative Analysis of DEGs and Differential Metabolites

To better understand the leaf coloring of red maple, we conducted a correlation analysis between the DEGs and DAMs mapped onto the flavonoid component biosynthesis pathway in the red and yellow strains. The Pearson correlation coefficients of the DEGs and DAMs were calculated using the R package. We constructed a gene–metabolite network map based on the Pearson correlation coefficients, with consideration of correlation coefficients > 0.9 or < −0.9 and *p*-value < 0.05, using Cytoscape v3.2.0.

**Figure 10:**
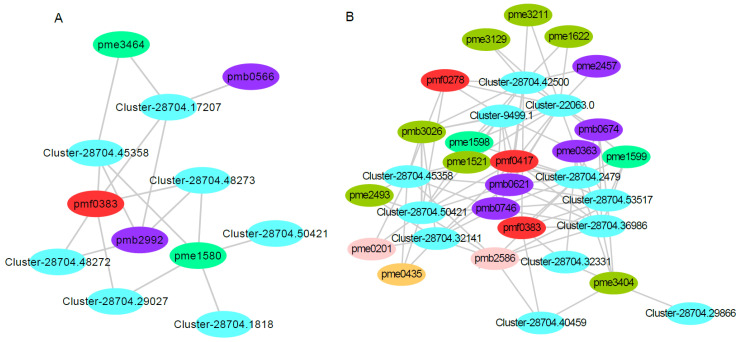
Correlation analysis of the transcriptomic and metabolomic data for red and yellow leaf coloring in red maple. Note: (**A**) the connection network between the DEGs and DAMs that mapped onto flavonoid metabolism in the red-strain red maple. Pmf0383, pmb2992, pme3464, pme1580, and pmb0566 are the metabolites of ‘5,7-dihydroxy-3′,4′,5′-trimethoxyflavone′, acacetin *O*-glucuronic acid, isosakuranetin (4′-Methylnaringenin), eriodictyol, and luteolin *O*-hexosyl-*O*-pentoside, respectively. (**B**) The connection network between the DEGs and DAMs that mapped onto flavonoid metabolism in the yellow-strain red maple. Pmb0621, pmb2586, pmb3026, pme1521, pme1598, pme1622, pme2457, pme3129, pme3211, pme3404, pmf0278, pmf0417, pmb0674, pmb0746, pme0201, pme0363, pme0435, pme1599, pme2493, and pmf0383 are the metabolites of C-hexosyl-isorhamnetin *O*-hexoside, Gallocatechin-catechin, Quercetin *O*-acetylhexoside, Dihydroquercetin (Taxifolin), Hesperetin 5-*O*-glucoside, Kaempferol 3-*O*-glucoside (Astragalin), Luteolin 7-*O*-glucoside (Cynaroside), Quercetin 4′-*O*-glucoside (Spiraeoside), Quercetin 3-*O*-glucoside (Isotrifoliin), Syringetin, Gossypitrin, Eriocitrin, C-pentosyl apigenin *O*-salicyloyl hexoside, Tricin 4′-*O*-β-guaiacylglycerol, Catechin, Chrysoeriol, Procyanidin B2, 7-*O*-Methyleriodictyol, Kaempferol 3,7-dirhamnoside (Kaempferitrin), and 5,7-Dihydroxy-3′,4′,5′-trimethoxyflavone, respectively.

Plant leaf coloring is related to flavonoid metabolism. Understanding the changes in gene and metabolite expression in the flavonoid content of the two red maple strains might explain some reasons for the red maple leaf coloring. Seven differentially expressed genes and five differentially accumulated metabolites were involved in the red strain of red maple (Figure 10A). The expression levels of Cluster-28704.45358 and Cluster-28704.50421, both encoding caffeoyl-CoA 3-O-methyltransferase (*CCOMT*), were up-regulated. Notably, these clusters catalyze caffeoyl-CoA to feruloyl-CoA. The expression levels of Cluster-28704.48272, Cluster-28704.48273, and Cluster-28704.29027, which encode dihydroflavonol 4-reductase (*DFR*), were up-regulated. These clusters catalyze the conversion of dihydroflavonols to leucoanthocyanidins in one of the last steps of anthocyanin biosynthesis. The gene encoding anthocyanidin reductase (*ANR*), Cluster-28704.17207, was up-regulated; this gene catalyzes the conversion of leucoanthocyanidin (flavan-3,4-diols) to epicatechin. The gene encoding shikimate O-hydroxycinnamoyltransferase (*HCT*), Cluster-28704.1818, was up-regulated; this gene catalyzes the conversion of caffeoyl quinic acid to caffeoyl-CoA. These results are consistent with previously reported results in other plants, and many structural genes, such as *CHS* [18,19], *DFR* [21,57], *PAL* [58], *CHI* [17,59], *4CL* [60,61,62], *F3H* [22,62,63], *ANS* [20], *CCOMT* [64], *ANR* [65], and *HCT* [66,67], participate in flavonoid metabolism. Additionally, a previous study also showed that 46 genes were related to anthocyanin biosynthesis in red maple [4]. These structural genes expressed in red maple were strongly correlated with flavonoid accumulation.

In addition, five differentially accumulated metabolites (pmf0383, pmb2992, pme3464, pme1580, and pmb0566) were all up-regulated. Pmf0383 (′5,7-dihydroxy-3′,4′,5′-trimethoxyflavone′) is a flavonoid; Pmb2992 (acacetin O-glucuronic acid) and pmb0566 (luteolin O-hexosyl-O-pentoside) are flavones; and pme3464 (isosakuranetin (4′-Methylnaringenin)) and pme1580 (eriodictyol) are flavanones (Figure 10A, Supplemental Table S5). Flavones and flavanones are the vital co-pigments responsible for leaf coloring, and they are also important for the leaf coloring of *Cymbidium sinense* [68].

In the yellow–red strain, 12 genes and 20 metabolites were involved in this pathway. Among these genes, Cluster-28704.45358 and Cluster-28704.50421, which encode caffeoyl-CoA 3-O-methyltransferase (*CCOMT*), were up-regulated. Two genes encode shikimate O-hydroxycinnamoyltransferase (HCT); one was up-regulated (Cluster-28704.32141), and the other was downregulated (Cluster-28704.42500). Cluster-28704.2479 and Cluster-28704.32331 encoding *CHS*, which catalyzes the synthesis of naringenin chalcone using p-coumaryl-CoA and malonyl-CoA, were down-regulated. Cluster-28704.29866 encoding ANS was down-regulated, which led to a reduction in anthocyanins. Cluster-28704.53517 encoding *CHI* was down-regulated; this cluster can regulate the content of chalcones and flavanones. The expression of Cluster-28704.40459 encoding *F3H*, which produces 3-hydroxylated flavonoids, was down-regulated. Cluster-9499.1 encoding flavonoid 3′,5′-hydroxylase (*F3′5′H*), which catalyzes the hydroxylation of the 3′ and 5′ ends of colorless dihydro flavonol B ring, was down-regulated. Cluster-22063.0 was down-regulated; this cluster encodes flavanone 7-O-glucoside 2′′-O-beta-L-rhamnosyltransferase (*F7GRT*), which catalyzes flavones and flavonols to become glucosylated in the C7 position. Cluster-28704.36986 was down-regulated; this cluster encodes trans-cinnamate 4-monooxygenase (*TC4M*), which catalyzes trans-cinnamate to 4-coumarate. In general, the DEGs involved in the flavonoid metabolic pathway in the red train red maple were up-regulated, while the corresponding DEGs in the yellow-strain red maple were down-regulated. Twelve differentially accumulated metabolites (pmb0621, pmb2586, pmb3026, pme1521, pme1598, pme1622, pme2457, pme3129, pme3211, pme3404, pmf0278, and pmf0417) were all down-regulated. Eight differentially accumulated metabolites (pmb0674, pmb0746, pme0201, pme0363, pme0435, pme1599, pme2493, and pmf0383) were up-regulated (Figure 10B, Supplemental Table S5). Moreover, Cluster-28704.45358 and Cluster-28704.50421 were up-regulated in the red strain but down-regulated in the yellow strain, and pmf0383 was up-regulated in both the red and yellow strains.

## 4. Conclusions

In our study, a module of leaf-coloring dynamic changes was built based on the phenotypic, metabolomic, and transcriptomic analyses of flavonoids′ metabolic processes and accumulation in red maple. The contents of total content of anthocyanin, chlorophyll-a, and chlorophyll-b, the total content of chlorophyll, and the component of flavonoids together determined the leaf color of red maple. The differentially expressed gene of caffeoyl-CoA 3-*O*-methyltransferase (*CCOMT*) (Cluster-28704.45358 and Cluster-28704.50421) was up-regulated in the red strain but down-regulated in the yellow strain, which might lead differences in the metabolite contents of anthocyanins, especially of Peonidin 3-*O*-glucoside chloride and Pelargonidin 3-*O*-beta-*D*-glucoside, flavone, and flavonol in the red and yellow strains. Additionally, these metabolites play essential roles in the leaf coloring of the red and yellow strains of red maple. For the complex metabolomics of flavonoids and the components of pigments in leaves, further research is needed to explore the mechanism of leaf coloring in red maple.

## Figures and Tables

**Table 1:** Statistical analyses and mapping results of RNA sequencing reads.

Sample	Raw Reads	Clean Reads	Clean Bases	Q20 (%)	Q30 (%)	GC Content (%)
AR1018y1	62,355,272	61,292,976	9.19G	97.12	92.14	43.34
AR1018y2	42,233,154	41,535,754	6.23G	96.38	90.43	43.11
AR1018y3	52,724,180	51,840,960	7.78G	97.02	92.06	43.23
AR1018r1	48,099,472	47,233,620	7.09G	97.12	92.03	43.22
AR1018r2	60,340,882	59,367,640	8.91G	96.86	91.70	43.08
AR1018r3	63,959,474	62,759,202	9.41G	97.05	92.10	43.21
AR1030y1	47,217,206	46,038,500	6.91G	97.06	92.13	43.04
AR1030y2	57,223,586	56,019,688	8.40G	97.02	91.82	42.96
AR1030y3	48,050,110	47,224,834	7.08G	97.10	91.95	43.29
AR1030r1	46,304,716	45,444,484	6.82G	97.41	92.59	43.05
AR1030r2	60,848,702	59,758,088	8.96G	96.96	91.68	43.04
AR1030r3	62,385,420	60,778,710	9.12G	97.23	92.26	43.03

## Data Availability

The data presented in this study are available in article and Supplementary Material.

## Supplementary Materials

The following supporting information can be downloaded at https://www.mdpi.com/article/10.3390/metabo13040464/s1. Figure S1. KEGG classification analysis of DAMs in different groups. Supplemental Table S1. The detected metabolite information among red maple leaves at different developmental stages. Supplemental Table S2. The differentially accumulated metabolites in different groups of red maple. Supplemental Table S3. The differentially expressed genes in different groups of red maple. Supplemental Table S4. The GO annotation of differentially expressed genes. Supplemental Table S5. Correlation analysis of the DEGs and DAMs for red and yellow leaf coloring in red maple.
