# Supplementary figures and images for: Integrative Transcriptomic and Metabolomic Analysis Reveals the Molecular Mechanism of Red Maple (Acer rubrum L.) Leaf Coloring
Source: Metabolites. 2023 Mar 23;13(4):464. doi: 10.3390/metabo13040464 (PMC10143518; doi:10.3390/metabo13040464)

AR1018r vs AR1031r

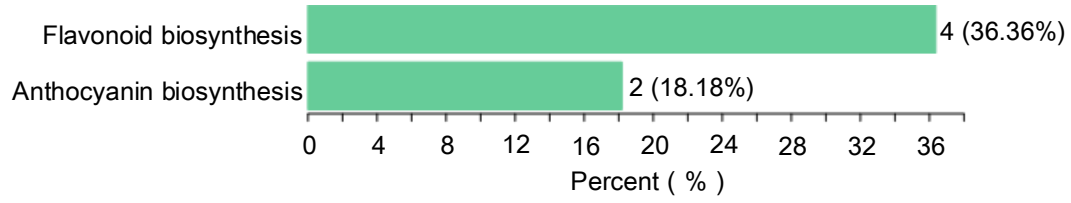

AR1018y vs AR1031y

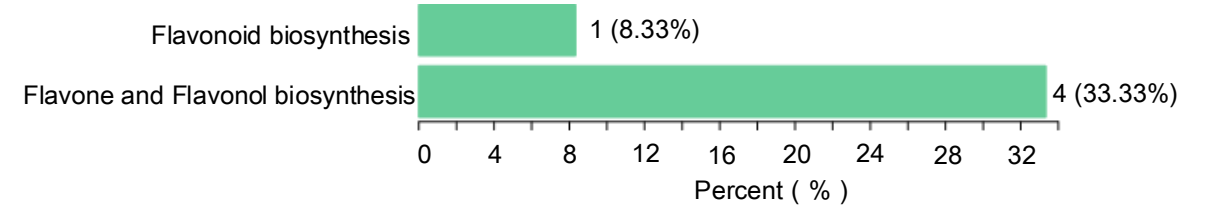

AR1018r vs AR1119r

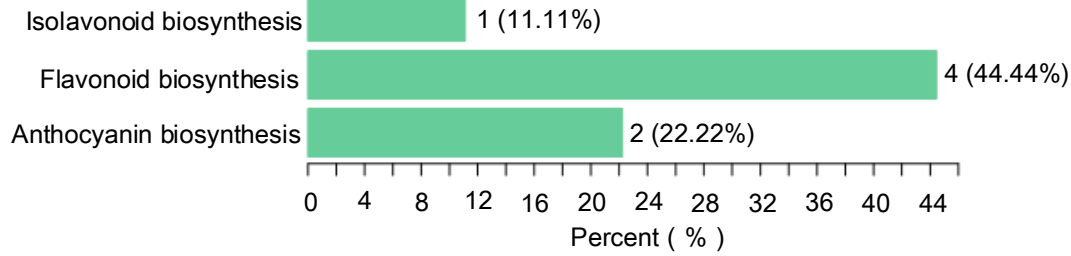

AR1018y vs AR1119y

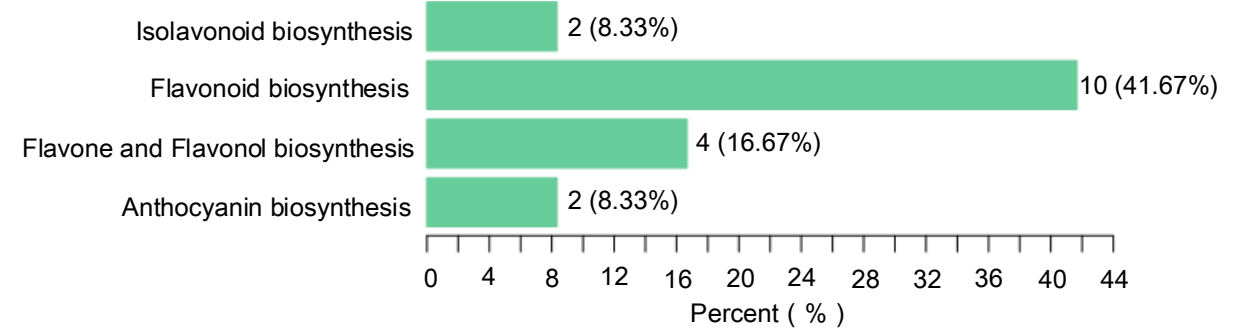

AR1031r vs AR1119r

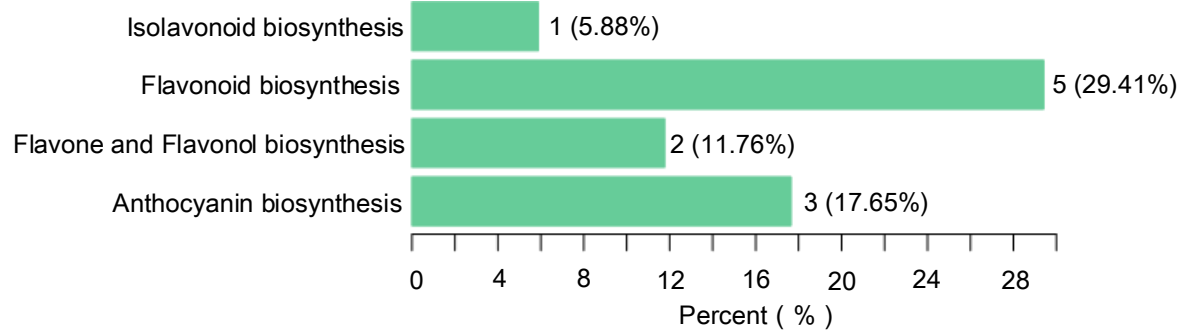

AR1031y vs AR1119y

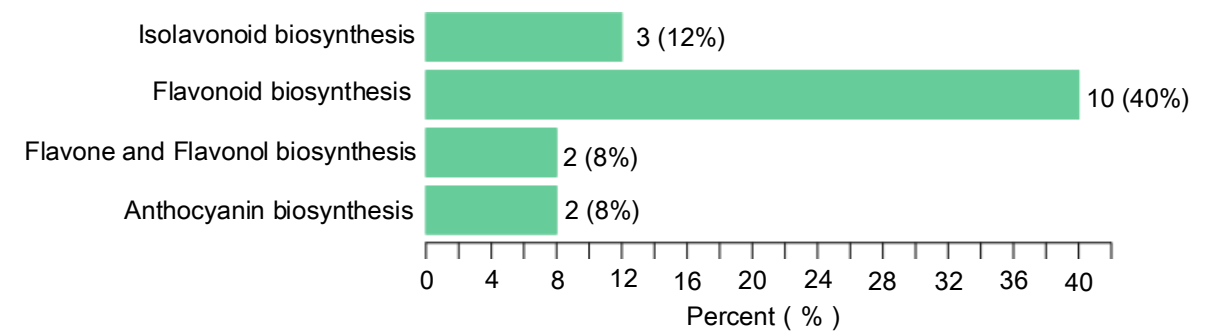

Supplement: Supplementary file 1 [file metabolites-13-00464-s001.zip › Figure S1.pdf]
